# A large-scale benchmark dataset for Urdu script optical character recognition with systematic augmentation

**DOI:** 10.1016/j.dib.2026.112938

**Published:** 2026-06-04

**Authors:** Fauzia Yasir, Majida Kazmi, Saad Ahmed Qazi

**Affiliations:** a Faculty of Electrical and Computer Engineering, NED University of Engineering and Technology, Karachi 75270, Pakistan; b Neurocomputation Lab, National Center of Artificial Intelligence, NED University of Engineering and Technology, Karachi 75270, Pakistan

**Keywords:** Low-resource language, Language digitization, OCR text images, Data annotation, Image augmentation, Natural language processing, Machine learning, Computer vision

## Abstract

Urdu is spoken by over 230 million people worldwide, yet it remains significantly underrepresented in digital resources, with limited availability of large-scale, publicly accessible training datasets for optical character recognition (OCR). The diversity of Urdu font styles encountered in printed books, newspapers, and digital publications poses a substantial barrier to developing generalizable OCR systems, while the absence of standardized benchmarks hinders fair and reproducible comparison across recognition approaches. This data article presents FIPU-OCR-CHAR, a benchmark dataset of printed Urdu characters encompassing 48 classes: 38 alphabets and 10 numerals in their isolated forms. The dataset was constructed through a fully systematic pipeline comprising five sequential stages: font collection and validation, character set definition, base image rendering, augmentation, and dataset organization with split generation. Each character class was rendered from 201 distinct Urdu TrueType/OpenType font files, producing 9,648 base images (201 fonts × 48 classes). Each base image was subsequently processed through 34 augmentation operations encompassing geometric transforms, photometric adjustments, blur filters, noise injection, and morphological operations, producing 328,032 augmented images. The complete dataset totals 337,680 labeled PNG images, each stored at 28×28 pixel resolution with 24-bit color depth. The dataset is organized into three predefined splits: training (70%; 236,376 images), validation (20%; 67,536 images), and testing (10%; 33,768 images), each accompanied by a CSV annotation file mapping image filenames to integer class labels (0–47). The repository additionally contains a Jupyter Notebook implementing a ResNet-34 baseline classification pipeline, a results summary image, and a README file documenting dataset structure and label definitions. The dataset is publicly available on Mendeley Data under a CC BY 4.0 license and is intended for use in OCR model development, font-invariant classifier training, Urdu script digitization, transfer learning for word- and line-level recognition, and benchmarking of convolutional neural network and Vision Transformer architectures on low-resource script character recognition tasks.

Specifications Table

**Table:** 

Subject	Computer Sciences
Specific subject area	Printed Urdu character recognition, OCR dataset, font-variant benchmark, deep learning, NLP, computer vision.
Type of data	Image (.png) Table (.csv label annotation files) Raw and systematically augmented synthetic character images.
Data collection	The dataset was constructed through a fully systematic pipeline by applying 201 distinct Urdu TrueType/OpenType font files to 48 character classes (38 alphabets + 10 numerals), producing 9,648 base images. Each base image was then subjected to 34 augmentation operations including rotation, shear, blur, noise injection, brightness/contrast variation, and morphological transforms to simulate printing and scanning artifacts, yielding 328,032 augmented images. All output images were resized to 28×28 pixels in PNG format with 24-bit depth. *.*
Data source location	Institution: NED University of Engineering and Technology City: Karachi Country: Pakistan*.*
Data accessibility	Repository name: Mendeley Data Data identification number: 10.17632/9cdk8y89v6.1 Direct URL to data: https://data.mendeley.com/datasets/9cdk8y89v6/1 Freely accessible; download Dataset.zip archive (approximately 162MB compressed); extract to access organized folder structure with train/validation/test splits; see README.txt for detailed structure and usage guidelines.
Related research article	F. Yasir and M. Kazmi, ``Acceleration of Urdu Optical Character Recognition on Zynq UltraScale+ MPSoC Using Deep Convolutional Neural Network,'' in IEEE Access, vol. 13, pp. 135538-135557, 2025, doi: 10.1109/ACCESS.2025.3593294

## 1 Value of the Data

• **Addresses a critical resource gap in Urdu OCR research.** Urdu is spoken by over 230 million people worldwide, yet publicly available, large-scale, font-diverse character-level datasets remain scarce. FIPU-OCR-CHAR fills this gap by providing 337,680 labelled images across 48 classes rendered in 201 distinct font styles, giving researchers a standardized, balanced, and reproducible foundation for developing and benchmarking Urdu OCR systems.
• **Enables font-invariant model training and evaluation.** The wide coverage of 201 font styles ensures that models trained on this dataset are exposed to the full typographic diversity encountered in real-world printed Urdu documents, digital publications, and archival materials. This diversity directly addresses the font-generalization challenge that limits the practical deployment of existing Urdu OCR systems.
• **Rich augmentation suite for robustness.** The 34 augmentation types applied to each base image simulate degradation scenarios such as scanning noise, blur, contrast variation, and geometric distortion. This makes the dataset directly applicable for training models intended to process degraded or low-quality printed Urdu text, such as scanned books, historical documents, and newspaper archives.
• **Standardized format enables direct integration with deep learning frameworks:** All images are standardized to 28×28 pixel resolution with consistent preprocessing, matching the widely adopted format for character recognition benchmarks (similar to MNIST, EMNIST). The dataset includes pre-split training (70%), validation (20%), and test (10%) sets with stratified sampling, comprehensive metadata in CSV format, and organized directory structure, enabling researchers to immediately train and evaluate deep learning models without additional preprocessing. The included ResNet-34 training notebook provides a reproducible baseline that can be directly extended or used as a benchmark reference.
• **Broad reuse potential across multiple tasks and architectures. T**he dataset supports transfer learning, script classification, font identification, data augmentation. It is compatible with standard deep learning frameworks (PyTorch, TensorFlow) and suitable for CNNs, Vision Transformers, and other image classification architectures.

## 2 Background

Urdu is the national language and a primary official language of Pakistan. It is also one of the official languages of India and is spoken by over 230 million people worldwide [1]. Despite this large speaker population, Urdu remains a low-resource language with limited digital resources and scarce large-scale datasets for optical character recognition (OCR) training [2]. Urdu characters evolved from a modified Perso-Arabic script in the 13th-century Indian subcontinent, blending Persian, Arabic, and Turkic influences with localized sounds. The cursive nature of Urdu script, derived from the Arabic writing system, presents unique challenges for OCR systems including context-dependent character shapes, ligature formation, and right-to-left text direction [3].

A few available Urdu character datasets for OCR research, including handwritten corpora such as CENPARMI (89,838 images) [4], UCOM (53,248 images, Nastaleeq only) [5], and UNHD (187,000 images, Nastaleeq only, 500 writers) [6], as well as the printed MMU-OCR-21 dataset (849 images, 3 font styles) [7]. However, these datasets are limited in scale, restricted predominantly to a single font style (Nastaleeq), and lack augmented training samples, collectively constraining the development of font-invariant OCR systems. The lack of comprehensive, font-diverse character-level datasets remains a significant bottleneck in advancing Urdu OCR technology, particularly for applications requiring robust performance across historical documents, contemporary publications, newspapers, books, and digitized archives [8]. This data article presents FIPU-OCR-CHAR, a large-scale, multi-font, annotated dataset of augmented Urdu character images designed to support font-invariant model development across diverse printed styles.

The dataset was created to support the research presented in the related IEEE Access article [9], which investigates hardware-accelerated deep learning for Urdu character recognition using a custom CNN architecture on Zynq UltraScale+ MPSoC. It serves as the training and evaluation foundation for font-invariant OCR models, enabling systematic investigation of deep learning architectures, augmentation strategies, and hardware acceleration techniques for Urdu text recognition.

## 3 Data Description

The repository hosted on Mendeley Data (DOI: 10.17632/9cdk8y89v6.1) contains four files: the main dataset archive (dataset.zip, 116 MB) along with detail documentation (README.md), a sample testing script (Urdu_OCR_Resnet_34_Sample_Code.ipynb) and its performance visualization (test_results.png).

Table 1 provides a comprehensive overview of the dataset organization.

**Table 1:** Dataset directory structure and organization.

Directory/File	Description	Contents
dataset.zip	Main dataset archive (116 MB)	Contains train, validate, and test folders
dataset/train/	Training set	236,375 images + train.csv
dataset/train/images/	Training images	PNG files (28×28, 24-bit)
dataset/train/train.csv	Training labels	2 columns: filepath, label (Urdu character)
dataset/validate/	Validation set	67,537 images + validate.csv
dataset/validate/images/	Validation images	PNG files (28×28, 24-bit)
dataset/validate/validate.csv	Validation labels	2 columns: filepath, label (Urdu character)
dataset/test/	Test set	33,768 images + test.csv
dataset/test/images/	Test images	PNG files (28×28, 24-bit)
dataset/test/test.csv	Test labels	2 columns: filepath, label (Urdu character)
README.txt	Dataset documentation	Usage instructions and dataset description
Urdu_OCR_Resnet_34_Sample_Code.ipynb	Sample testing script	Python code for ResNet-34 model evaluation
test_results.png	Performance visualization	Text output

### 3.1 Dataset.zip

This the primary data archive. Once extracted, it yields three split directories: train/, val/, and test/, each containing an images/ subfolder and a CSV annotation file. The overall dataset composition is summarized in Table 1.

• train/ : This folder contains 70% of all images (236,375 images) organized into 48 class subfolders, along with a train.csv annotation file mapping each image filename to its corresponding Urdu character class label.
• val/ : This folder contains 20% of all images (67,537 images) with the same subfolder structure and a val.csv annotation file.
• test/ : This folder contains 10% of all images (33,768 images) with a test.csv annotation file.

### 3.2 File naming convention

The dataset employs a systematic naming scheme that encodes each image's type, source, and augmentation history. Each character class is represented by two image types: base images capturing font style variation alone, and augmented images incorporating additional transformations applied over each base image.

Base images follow the convention {base_id}.png, where base_id ranges from 1 to 9,648 (48 character classes × 201 font files). Base images are indexed sequentially, where all 201 font variants of a character class are assigned consecutive ids before advancing to the next character class (e.g., class 1 spans ids 1–201, class 2 spans ids 202–402, and so on through class 48 ending at id 9,648).

Augmented images follow the convention {base_id}-{aug_id}.png, where base_id identifies the source base image (1–9,648) and aug_id identifies the augmentation operation applied (1–34). For example, 1-1.png refers to the first augmentation operation applied to base image 1, while 9648-34.png refers to the last augmentation operation applied to the last base image. All images are distributed across the three split folders alongside their corresponding CSV annotation files. Table 2 illustrates the naming convention with representative examples.

**Table 2:** File naming convention examples.

Filename	Type	Description
1.png	Base image	Class 1 (first character), Font style 1 (The first font variant)
201.png	Base image	Class 1 (first character), Font style 201 (The last font variant)
202.png	Base image	Class 2 (second character), Font style 1 (The first font variant)
9648.png	Base image	Class 48 (last character), Font style 201 (last base image in dataset)
1-1.png	Augmented image	1.png with augmentation operation 1
1-34.png	Augmented image	1.png with augmentation operation 34
9648-34.png	Augmented image	9648.png with augmentation operation 34

### 3.3 CSV annotation files

Each split includes a CSV annotation file providing the mapping between image filenames and their corresponding Urdu character labels. The CSV files are comma-delimited(,) and use UTF-8 encoding to properly represent Urdu characters, ensuring compatibility across different operating systems and programming environments. Each row corresponds to one image, with a filepath column providing the relative path from the split directory and a label column containing the ground-truth Urdu character. The CSV files are designed for seamless integration with standard machine learning frameworks including PyTorch, TensorFlow, and scikit-learn. Table 3 summarizes the statistics of each annotation file.

**Table 3:** CSV file statistics.

Split	CSV Filename	Number of Rows	Approximate File Size
Train	**train.csv**	236,375	10 MB
Validation	**validate.csv**	67,537	3 MB
Test	**test.csv**	33,768	1.5 MB

Table 4 presents representative sample rows from CSV annotation files, where filepath = relative path from split root, label = ground-truth Urdu character in UTF-8 Unicode. Table 5 provides the complete label-to-character mapping for all 48 classes. Integer indices correspond to label values in CSV annotation files.

**Table 4:** Sample rows from the train.csv annotation file.

Filepath	label
./images/3743-28.png	ج
./images/6535-16.png	ث
./images/1162-14.png	ی
./images/9281-11.png	ظ
./images/7050-28.png	ص

**Table 5:** Label to character mapping (0-47).

Idx	Character	Unicode	Idx	Character	Unicode	Idx	Character	Unicode	Idx	Character	Unicode
0	0	U+0030	12	ب	U+0628	24	ص	U+0635	36	ٹ	U+0679
1	1	U+0031	13	ت	U+062A	25	ض	U+0636	37	پ	U+067E
2	2	U+0032	14	ث	U+062B	26	ط	U+0637	38	چ	U+0686
3	3	U+0033	15	ج	U+062C	27	ظ	U+0638	39	ڈ	U+0688
4	4	U+0034	16	ح	U+062D	28	ع	U+0639	40	ڑ	U+0691
5	5	U+0035	17	خ	U+062E	29	غ	U+063A	41	ژ	U+0698
6	6	U+0036	18	د	U+062F	30	ف	U+0641	42	ک	U+06A9
7	7	U+0037	19	ذ	U+0630	31	ق	U+0642	43	گ	U+06AF
8	8	U+0038	20	ر	U+0631	32	ل	U+0644	44	ھ	U+06BE
9	9	U+0039	21	ز	U+0632	33	م	U+0645	45	ی	U+06CC
10	ء	U+0621	22	س	U+0633	34	ن	U+0646	46	ے	U+06D2
11	ا	U+0627	23	ش	U+0634	35	و	U+0648	47	آ	U+0622

### 3.4 Character classes and distribution

The dataset encompasses 48 character classes representing the complete Urdu writing system: 38 alphabetic characters and 10 numerals (0–9). Table 6 presents the complete character inventory with Unicode representations.

**Table 6:** Urdu character classes in FIPU-OCR-CHAR dataset.

Each character class contains 34 variants per font style: one base image representing the character in that font style without any transformation, and 34 augmented images generated by applying distinct augmentation operations to that base image. Since the dataset encompasses 201 distinct Urdu font styles, each character class is represented by a total of 7,035 images (201 font styles × 34 variants), ensuring uniform class balance across the entire dataset. The stratified splitting strategy maintains this balance across all three splits, with each character class proportionally represented in training (70%), validation (20%), and testing (10%) sets.

### 3.5 Image specifications

All images in the dataset adhere to the standard specifications required for training and testing the deep learning models for OCR application. Table 7 details the technical properties of the image files. The 28×28 pixel resolution was selected to align with established character recognition benchmarks such as MNIST [10] and EMNIST [11], facilitating comparative analysis and transfer learning. The 24-bit RGB color depth ensures compatibility with standard deep learning frameworks and image processing libraries. To prevent downscaling-induced interpolation artifacts and preserve critical stroke-width details, all images were natively rendered at 28×28 pixels using the Pillow FreeType-based renderer at 12pt. PNG lossless compression preserves character details while maintaining manageable file sizes, with the complete dataset totalling 116 MB in compressed form.

**Table 7:** Image technical specifications.

Property	Value
File Format	PNG (Portable Network Graphics)
File Size	250-350 bytes per image
Image Dimensions	28 × 28 pixels
Width	28 pixels
Height	28 pixels
Horizontal Resolution	96 DPI
Vertical Resolution	96 DPI
Bit Depth	24-bit (8 bits per channel)
Color Space	RGB
Compression	PNG lossless compression
Background Color	White (255, 255, 255)
Foreground Color	Black (0, 0, 0)
Total Dataset Size	116 MB (compressed in ZIP)

### 3.6 Font diversity and coverage

The dataset incorporates 201 distinct Urdu and Arabic-script fonts sourced from publicly available and font repositories. The collection spans 17 font families and series alongside 45 standalone typefaces, covering the full typographic range used in Urdu print media, digital publishing, and religious texts. Table 8 summarizes the font families included.

**Table 8:** Font families in FIPU-OCR-CHAR.

Font Family / Series	Count	Variants / Notes
XB Series	60	13 sub-families: Kayhan, Khoramshahr, Niloofar, Riyaz, Roya, Shafigh (incl. Kurd & Uzbek), Shiraz, Sols, Tabriz, Titre, Yagut, Yas, Zar
Individual Fonts	45	Standalone typefaces including Faiz Lahori Nastaleeq, Iran Nastaliq, Nafees Nastaleeq, Layla Ruqaa, PakType Naskh Basic, Lateef, Mehr Nastaliq Web, among others
Al-Qalam Collection	12	Alvi Nastaleeq, Farhan, Fawad (Bold/Light), Istiaq, Kolkatta Quranic, Makki, Nabeel, Punjbi, Quran 2A, Taj Nastaleeq, Telenor
XM Series	12	Traffic (x4), Vahid (x4), Yermook (x4)
Noto Kufi Arabic	9	x9 weights: Thin through Black
XP Series	8	Vosta (x4), Ziba (x4)
Baloo Bhaijaan 2	5	Regular, Medium, Semi Bold, Bold, Extra Bold
AA Sameer	4	Armaa, Kelk, Qamri, Zikran
Adobe Arabic	4	Regular, Italic, Bold, Bold Italic
Jameel Noori	4	Kasheeda, Nastaleeq, Nastaleeq Kasheeda, Nastaleeq Regular
MJ Series	4	Dinar Two Light, Dinar Two Medium, Faten, Free
Mothanna	4	Regular, Bold, Oblique, Bold Oblique
Noto Nastaliq Urdu	4	Regular, Medium, Semi Bold, Bold
AlFars	3	Kamran Bold, Kodak, Badr
PDMS	3	Bukhari, Jauhar, Mehran
XW Series	2	Zar It, Zar It Bold
Smaller families (2 fonts each)	16	Alvi (x2), Asvcodar (x2), Badr (x2), DecoType (x2), Hassan (x2), Jalal (x2), Maged (x2), Mitra (x2), Nazanin (x2)
**Total**	**201**	

**Fig. 1:**
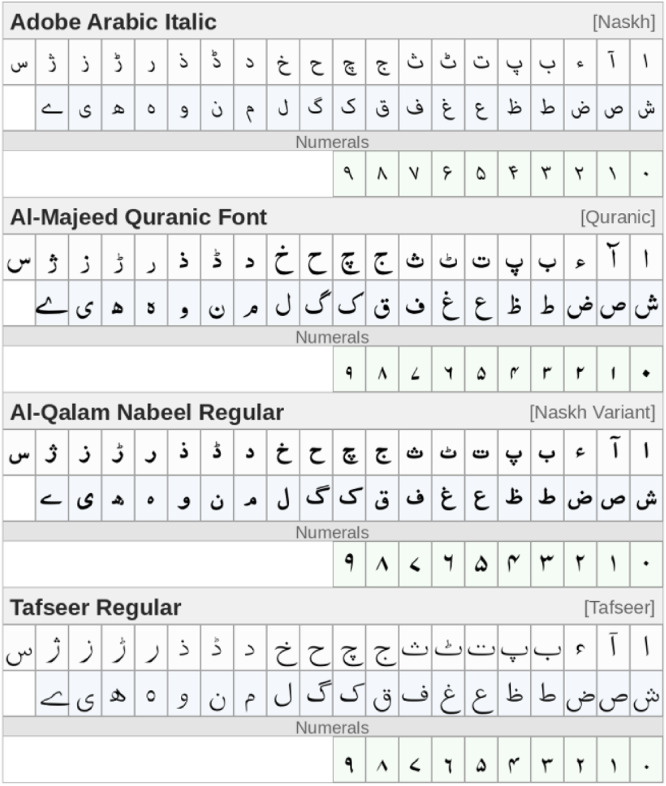
Representative renderings of all 48 character classes across four calligraphic styles, sampled from the 201 font styles in FIPU-OCR-CHAR, illustrating typographic variation across the dataset.

The collection represents five major calligraphic styles used in Urdu and Arabic typography, with representative character renderings across selected font styles illustrated in Fig. 1. The Nastaleeq style: the dominant script form for Urdu is the most extensively represented, with fonts including Jameel Noori Nastaleeq, Nafees Nastaleeq, Alvi Nastaleeq, Mehr Nastaliq Web, Noto Nastaliq Urdu, Iran Nastaliq, Faiz Lahori Nastaleeq, Fajer Noori Nastalique, Urdu Emad Nastaleeq, and Nastaleeq Like. The Naskh style is represented by PakType Naskh Basic and the XB, XM, and XP series. Kufi style is covered by the full Noto Kufi Arabic weight range. Thuluth style is represented by DecoType Thuluth, and Ruqaa by Layla Ruqaa. Font weight distribution across the collection comprises Regular (largest group), Bold, Italic, Bold Italic, Light, Semi Bold, Medium, Extra Bold, and specialized weights including Thin, Black, Extra Light, and Oblique variants.

1. Urdu_OCR_Resnet_34_Sample_Code.ipynb.

This file provides a complete end-to-end pipeline including data loading and preprocessing, a ResNet-34 model implementation, training and evaluation loops with validation and test accuracy metrics, and sample prediction visualizations. Key training parameters defined in the notebook include:

• Batch size: 256.
• Input resolution: 64×64 pixels.
• Epochs: 3.
• Learning rate: 1×10⁻⁴.
• Top-K predictions: 3.
• Training augmentation: RandomRotation(±15°), RandomAffine (translate=0.1, scale=0.9–1.1), and ColorJitter (brightness=0.2, contrast=0.2).

Upon execution, the notebook produces training and validation accuracy curves (train_val_accuracy.png) and top-3 test predictions with confidence scores (test_predictions.csv). It is intended as a reproducible baseline and can be adapted for alternative architectures. Complete baseline specifications, evaluation metrics, and performance analysis are provided in the Baseline Model Validation subsection of the Experimental Design section.

2. test_results.png.

This image file contains the output generated upon executing Urdu_OCR_Resnet_34_Sample_Code.ipynb, providing a visual record of the baseline model's training and evaluation run. It serves as a reference to verify expected notebook output and to confirm reproducibility when re-running the pipeline on the dataset.

3. README.txt.

The document explain the dataset structure, class label definitions, instructions for downloading and extracting the archive, guidance on running the training notebook, and citation information.

## 4 Experimental Design, Materials and Methods

**Fig. 2:**
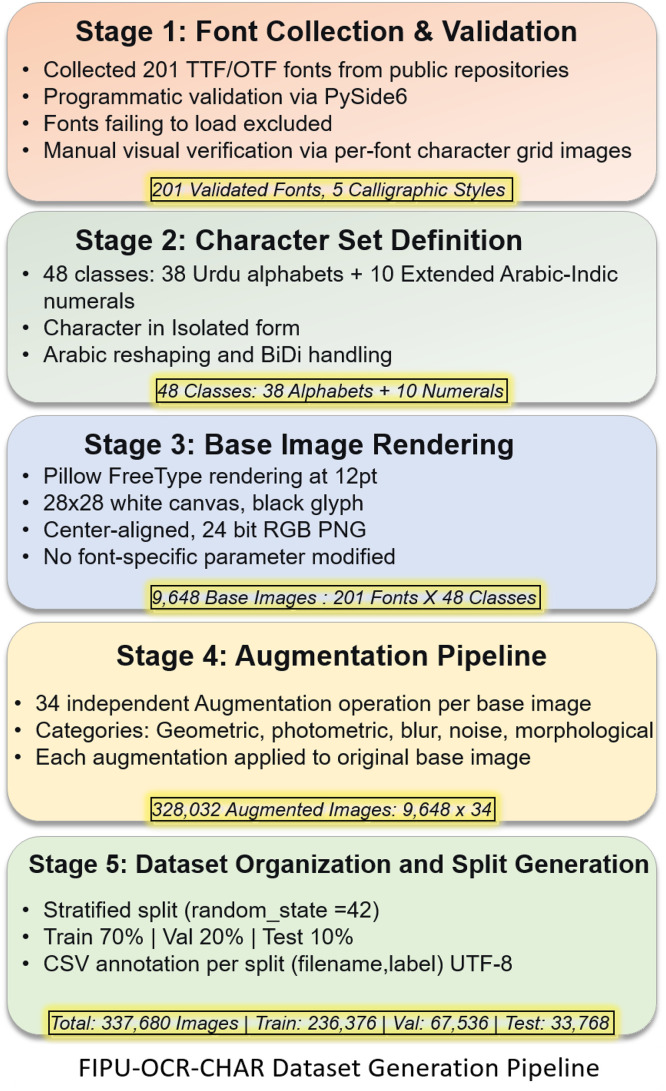
Overview of the FIPU-OCR-CHAR dataset generation pipeline. The process proceeds through five sequential stages from font style validation to final dataset organization.

The dataset was constructed through a systematic framework software-driven pipeline implemented in Python 3.10, utilizing a multi-library stack based on PySide6 [12] for font validation, Pillow [13] for base image rendering, Albumentations for augmentation operations and OpenCV for morphological processing. The pipeline consists of five sequential stages: font collection and validation, character set definition, base image rendering, augmentation, and dataset organization with split generation. The detail steps of proposed methodology are shown in Algorithm 1. Fig. 2 illustrates the overall workflow.

**Algorithm 1:** FIPU-OCR-CHAR dataset generation pipeline.

**Input:** Font directory F = {f₁, f₂, ..., f₂₀₁}
Character set C = {c₁, c₂, ..., c₄₈}
Augmentation set A = {a₁, a₂, ..., a₃₅}
**Output:** Dataset D with train/val/test splits
**Step 1 — Font Validation:**
For each font fᵢ in F:
Load fᵢ using PySide6 Qt-based font rendering library
If fᵢ fails to load or resolves no font family → discard
Else → add to validated set F'
For each font fᵢ in F':
Generate character grid image (48 chars, 66pt, 180×180px cells)
Inspect grid manually for glyph coverage and rendering quality
If font fails visual inspection → discard from F'
Result: |F'| = 201 validated fonts
**Step 2 — Character Set Definition:**
Define C = 38 Urdu alphabets + 10 Extended Arabic-Indic numerals
Apply Arabic text reshaping and bidirectional handling to all cⱼ
Result: |C| = 48 character classes in isolated form
**Step 2 — Base Image Generation:**
base_id ← 1
For each character cⱼ in C:
For each font fᵢ in F':
Render cⱼ using fᵢ on 28×28 white canvas at 12pt Centre-align glyph, black foreground
Save as {base_id}.png with label j
base_id ← base_id + 1
Result: 9,648 base images (48 classes × 201 fonts)
**Step 3 — Augmentation:**
For each base image {base_id}.png:
For k = 1 to 34:
Apply augmentation a_k_ with parameters in Table 9
Save as { base_id}-{k}.png
Result: 328,032 augmented images (9,648 × 34)
**Step 4 — Dataset Organization and Split Generation:**
Apply stratified split (random_state=42):
Train ← 70% | Val ← 20% | Test ← 10%
For each split:
Move images to respective folder
Generate CSV annotation per split:
columns: [filepath, label]
encoding: UTF-8
Result: 337,680 total images
Train: 236,376 | Val: 67,536 | Test: 33,768

### 4.1 Stage 1: Font collection and validation

The pipeline was initiated by assembling and validating the font collection that would serve as the typographic basis for all subsequent image generation. TrueType font files (.ttf) were collected from publicly available Urdu font repositories. Font selection prioritized three criteria: Unicode compatibility with the Urdu character block and Extended Arabic-Indic digit range; glyph legibility at the target 28×28 pixel resolution; and coverage of calligraphic styles and weight variants representative of real-world Urdu printing across books, newspapers, and digital publications. TrueType format was selected for its near-universal support across operating systems and rendering engines, ensuring consistent cross-platform reproducibility.

Each collected font was programmatically validated using a Qt-based font rendering library [12]. Fonts that failed to load or yielded no resolvable font family were automatically excluded from the collection. This validation step ensured that all fonts confirmed renderable before image generation commenced, preventing corrupt or blank images from entering the dataset. Following programmatic validation, manual visual verification was performed by reviewing per-font character grid images generated using PySide6, displaying all 48 characters rendered at 66pt within dedicated 180×180 pixel cells arranged in a grid of 20 characters per row. This verification confirmed consistent glyph coverage and rendering quality across all fonts. The final validated collection comprised 201 fonts.

### 4.2 Stage 2: Character set definition

In this step, the target character set was defined by reviewing standard Urdu orthography and identifying the complete set of printed Urdu characters encountered in real-world documents. The dataset encompasses 48 character classes: 38 alphabets and 10 numerals. The 38 alphabets cover both base Arabic letters shared across Arabic-script languages and Urdu-specific extensions that distinguish Urdu from Arabic and Persian. Characters were defined in their isolated forms, as isolated-form representation provides unambiguous visual boundaries between classes and constitutes the standard starting point for character-level recognition benchmarks.

The 10 numerals were rendered in their Extended Arabic-Indic printed forms (۰–۹) as produced by the Urdu font files, reflecting standard in printed Urdu books, newspapers, and official documents. To ensure correct Unicode shaping and right-to-left text directionality during rendering, all characters were processed through Arabic text reshaping and bidirectional text handling prior to rendering. In the CSV annotation files, these numeral classes are identified using Western Arabic-Indic digit labels (0–9, U+0030–U+0039) for compatibility with standard machine learning frameworks.

### 4.3 Stage 3: Character image rendering

Following font collection and character set definition, base image generation was performed using Pillow, a Python image processing library providing FreeType-based TrueType font rendering. For each validated font, a Font object was instantiated at 12pt with anti-aliasing enabled to produce smooth, clean and consistent glyphs suitable for robust pattern recognition. An Image canvas was then initialized with a white background (RGB: 255, 255, 255) of 28×28 pixels and each character was drawn at centre alignment. The black foreground on white background convention follows established character recognition benchmarks such as MNIST and EMNIST, facilitating direct comparability and transfer learning applicability. Pillow applies consistent anti-aliasing across all TrueType font files via the FreeType backend, ensuring uniform glyph rendering quality regardless of font style. No font-specific rendering parameters were modified, thereby preventing differential intensity gradients across the dataset.

This procedure produced the 9,648 base images (201 font styles × 48 character classes). Automated rendering from digital font files ensures pixel-level consistency in background color, canvas dimensions, and glyph positioning across all base images, such that typographic variation across the 201 font styles remains the sole source of visual diversity in the base dataset.

### 4.4 Stage 4: Augmentation pipeline

Data augmentation was applied to the 9,648 base images produced in the previous stage to expose models to the range of distortions encountered in real-world printed and scanned Urdu documents, including degradation from aging, photocopying, scanning noise, and geometric distortion. Each base image was independently subjected to 34 augmentation operations using Python image processing libraries including Albumenatations (v1.3.1) and OpenCV (v4.10.0.84), producing one augmented variant per operation per base image. Each augmentation was applied to the original base image independently rather than chained sequentially, ensuring each augmented image represents a single controlled distortion type. All augmentations operations used fixed, deterministic parameter ranges as specified in Table 9. This stage produced 328,032 augmented images (34 × 9,648).

**Table 9:** Augmentation operations applied to each base image along with parameter specifications.

No.	Augmentation	Category	Library & Parameters
1	Brightness & Contrast Low	Photometric	Albumentations RandomBrightnessContrast(brightness_limit=(-0.1,0.1), contrast_limit=(-0.1,0.1))
2	Brightness & Contrast Medium	Photometric	Albumentations RandomBrightnessContrast(brightness_limit=(-0.3,0.3), contrast_limit=(-0.3,0.3))
3	Brightness & Contrast High	Photometric	Albumentations RandomBrightnessContrast(brightness_limit=(-0.5,0.5), contrast_limit=(-0.5,0.5))
4	Brightness Only	Photometric	Albumentations RandomBrightnessContrast(brightness_limit=(-0.5,0.5), contrast_limit=(0,0))
5	Contrast Only	Photometric	Albumentations RandomBrightnessContrast(brightness_limit=(0,0), contrast_limit=(-0.5,0.5))
6	CLAHE Low	Photometric	Albumentations CLAHE(clip_limit=2.0, tile_grid_size=(8,8))
7	CLAHE High	Photometric	Albumentations CLAHE(clip_limit=4.0, tile_grid_size=(8,8))
8	RGB Shift Light	Photometric	Albumentations RGBShift(r/g/b_shift_limit=10)
9	RGB Shift Medium	Photometric	Albumentations RGBShift(r/g/b_shift_limit=20)
10	RGB Shift High	Photometric	Albumentations RGBShift(r/g/b_shift_limit=30)
11	RGB Shift Extreme	Photometric	Albumentations RGBShift(r/g/b_shift_limit=40)
12	Hue Saturation Low	Photometric	Albumentations HueSaturationValue(hue=10, sat=20, val=10)
13	Hue Saturation High	Photometric	Albumentations HueSaturationValue(hue=30, sat=50, val=30)
14	Sepia	Photometric	Albumentations ToSepia() default parameters
15	Blur	Filter	Albumentations Blur(blur_limit=3)
16	Sharpen Light	Filter	Albumentations Sharpen(alpha=(0.1,0.2), lightness=(0.5,0.6))
17	Sharpen Heavy	Filter	Albumentations Sharpen(alpha=(0.4,0.6), lightness=(0.7,1.0))
18	JPEG Compression Low	Compression	Albumentations ImageCompression(quality_lower=70, quality_upper=90)
19	JPEG Compression High	Compression	Albumentations ImageCompression(quality_lower=30, quality_upper=50)
20	Emboss Light 1	Texture	Albumentations Emboss(alpha=(0.1,0.2), strength=(0.2,0.3))
21	Emboss Light 2	Texture	Albumentations Emboss(alpha=(0.2,0.3), strength=(0.3,0.4))
22	Emboss Medium	Texture	Albumentations Emboss(alpha=(0.3,0.5), strength=(0.4,0.6))
23	Elastic Transform	Geometric	Albumentations ElasticTransform(alpha=0.2, sigma=5, alpha_affine=3)
24	Optical Distortion Light	Geometric	Albumentations OpticalDistortion(distort_limit=(0.1,0.2), shift_limit=(0.01,0.02))
25	Optical Distortion Medium	Geometric	Albumentations OpticalDistortion(distort_limit=(0.2,0.5), shift_limit=(0.02,0.05))
26	Optical Distortion Heavy	Geometric	Albumentations OpticalDistortion(distort_limit=(0.5,0.8), shift_limit=(0.1,0.1))
27	Optical Distortion Negative	Geometric	Albumentations OpticalDistortion(distort_limit=(-0.5,-0.2), shift_limit=(-0.05,-0.02))
28	Optical Distortion Shift Only	Geometric	Albumentations OpticalDistortion(distort_limit=(1.0,1.5), shift_limit=(0.05,0.1))
29	Optical Distortion Linear	Geometric	Albumentations OpticalDistortion(distort_limit=(0.2,0.5), shift_limit=(0.02,0.05), interpolation=INTER_LINEAR)
30	Optical Distortion Border Constant	Geometric	Albumentations OpticalDistortion(distort_limit=(0.2,0.5), shift_limit=(0.02,0.05), border_mode=BORDER_CONSTANT)
31	Shift Scale Rotate/Affine	Geometric	Albumentations composite: scale±0.25, shift±0.1, rotate±2 deg, shear±5 deg
32	Pixel Dropout Black	Noise	Albumentations PixelDropout(default dropout_prob, drop_value=0)
33	Pixel Dropout White	Noise	Albumentations PixelDropout(dropout_prob=0.1, drop_value=255)
34	Erosion	Morphological	OpenCV v4.10.0.84: cv2.erode(kernel=ELLIPSE(3×3), iterations=1)

### 4.5 Stage 5: Dataset organization and split generation

Following augmentation, the complete dataset of 337,680 images was partitioned into training (70%), validation (20%), and testing (10%) sets. This split was executed using scikit-learn’s train_test_split using stratified random sampling (stratify=label, random_state=42) to ensure that each of the 48 character classes maintained proportional representation across all subsets. Splitting was performed at the image level by character class label. The resulting data were organized into dedicated directories (train/, val/, and test/), each containing an images/ subfolder and a corresponding CSV annotation file. UTF-8 encoding was employed for these annotations to guarantee the accurate storage and cross-platform compatibility of Urdu Unicode characters. Within each CSV file, rows map the relative image filepaths to their respective integer class labels (0–47).

### 4.6 Baseline model validation

To demonstrate dataset usability and provide a reproducible performance reference, a ResNet-34 baseline model was trained and evaluated on FIPU-OCR-CHAR.

**Fig. 3:**
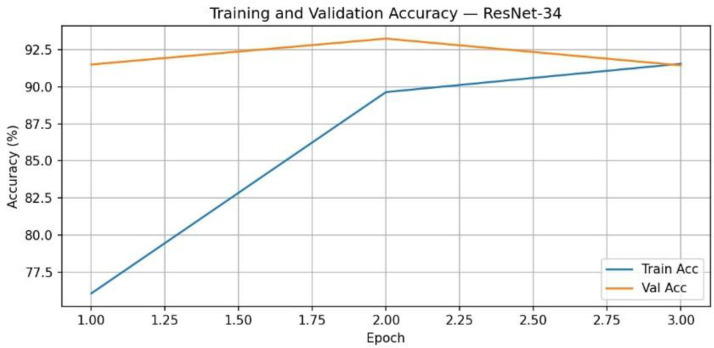
Training and validation accuracy curve.

The model was implemented using PyTorch 2.4.0 and torchvision 0.19.0 with CUDA 12.1 support on Python 3.10 with a pretrained ResNet-34 backbone with ImageNet weights was used, with the final fully-connected layer replaced to support 48-class classification. Input images were resized to 64×64 pixels prior to inference. Training was performed using the Adam optimizer with a learning rate of 1×10⁻⁴ and CrossEntropyLoss, a batch size of 256, and 3 epochs. A ReduceLROnPlateau scheduler (mode=max, factor=0.5, patience=3) was applied and the model checkpoint achieving the highest validation accuracy across all epochs was retained for final test evaluation. No explicit regularization was applied. The complete training configuration is summarized in Table 10, and per-epoch progression and evaluation metrics are presented in Table 11. Training and validation accuracy curves are shown in Fig. 3.

**Table 10:** ResNet-34 baseline training configuration.

Parameter	Value
Framework	PyTorch 2.4.0, torchvision 0.19.0, CUDA 12.1, Python 3.10
Model	ResNet-34 (ImageNet pretrained; FC layer replaced for 48-class output)
Input resolution	64×64 pixels (upscaled from 28×28)
Batch size	256
Epochs	3 (best model selected by validation accuracy)
Optimizer	Adam (lr = 1e-4)
LR Scheduler	ReduceLROnPlateau (mode=max, factor=0.5, patience=3)
Loss function	CrossEntropyLoss
Regularization	None (no dropout or weight decay)
Training augmentation	RandomRotation(±15 deg), RandomAffine(translate=0.1, scale=0.9-1.1), ColorJitter(brightness=0.2, contrast=0.2)
Data shuffling	Training set shuffled per epoch; val and test sets fixed
Hardware	CUDA GPU if available, CPU otherwise
Normalization	mean=[0.5, 0.5, 0.5], std=[0.5, 0.5, 0.5]

**Table 11:** Per-epoch training log. Best model at Epoch 2(val_acc=93.25%).

Epoch	Train Loss	Train Accuracy	Validation Accuracy
1	0.7664	76.06%	91.50%
2	0.3020	89.64%	93.25% (Best model saved)
3	0.2447	91.55%	91.44%

**Fig. 4:**
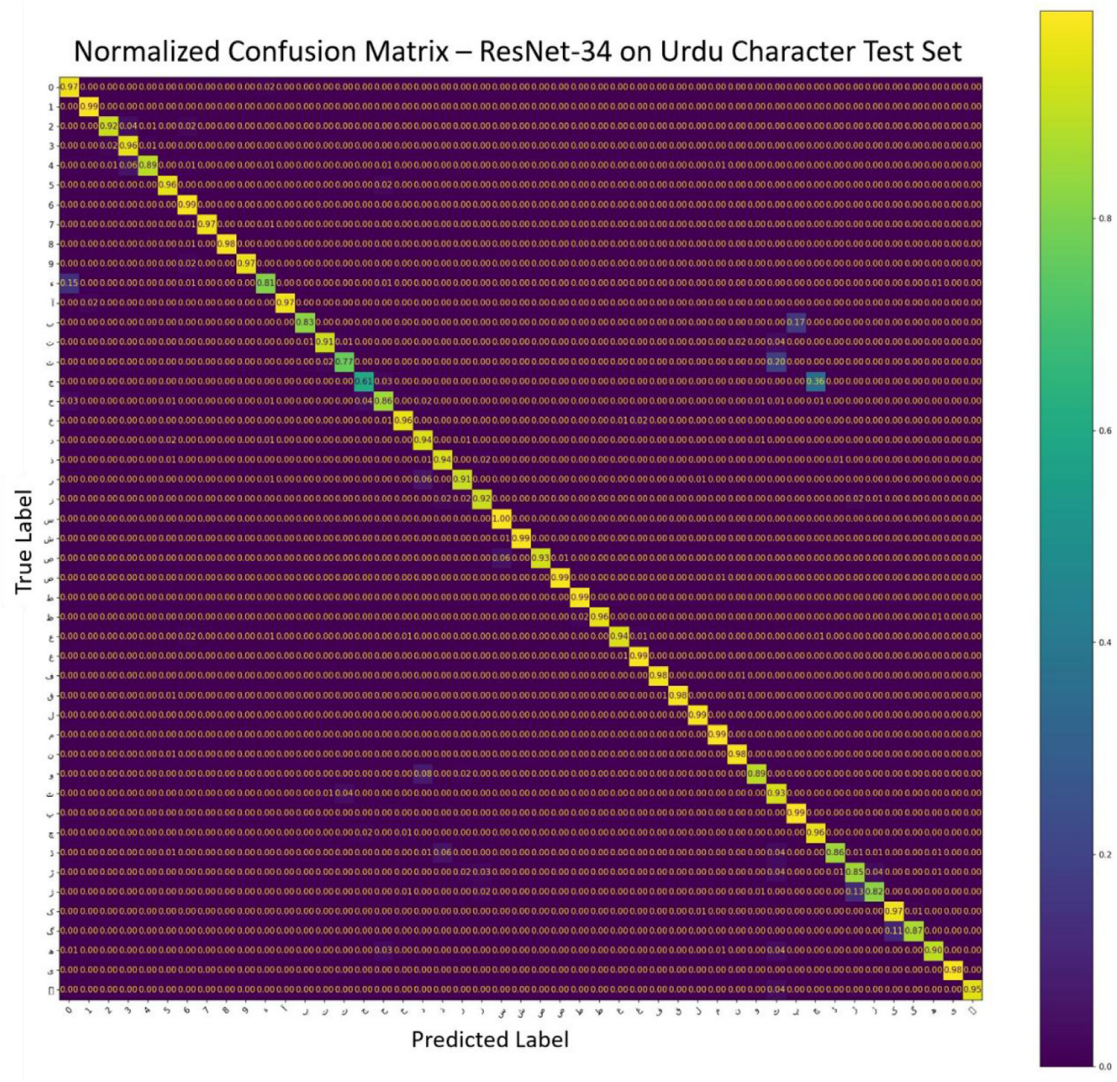
Normalized confusion matrix.

Summary evaluation metrics are presented in Table 12 and the normalized confusion matrix in Fig. 4. The three highest confusion rates  (35.51%),  (19.60%), and  (16.76%) which involve character pairs that share identical base strokes and differ only in diacritic mark. These confusions are visually attributable to the structural similarity between diacritic-differentiated character pairs, where the distinguishing feature such as the number and placement of dots occupies only a small portion of the 28×28 pixel canvas and may be further obscured by augmentation operations such as Gaussian blur, noise injection, and morphological erosion, which disproportionately affect fine dot structures relative to base strokes. This suggests that augmentation operations targeting high-frequency components warrant careful parameterization when applied to diacritic-rich scripts such as Urdu.

**Table 12:** Summary evaluation metrics of the ResNet-34 baseline on the test set(33,088 images, 48 classes).

Metric	Value
Final Test Accuracy (Top-1)	93.04%
Top-3 Accuracy	99.22%
Top-5 Accuracy	99.69%
Balanced Accuracy	0.9304
Macro F1-Score	0.9303
Weighted F1-Score	0.9303
Macro Average Precision (AP)	0.9811
Micro Average Precision (AP)	0.9826

To mitigate these error patterns, future architectures trained on this dataset may benefit from attention mechanisms that explicitly weight diacritic regions, multi-scale feature extraction to preserve fine-grained dot structure at low resolutions, or diacritic-aware loss functions that penalize confusion between structurally similar character pairs more heavily than dissimilar ones. These findings are intended to actively guide architectural design choices for low-resource Urdu and similar script OCR research. The baseline results confirm that FIPU-OCR-CHAR supports effective training of deep learning models for Urdu character recognition and serves as a reproducible benchmark reference for evaluating future architectures on this dataset.

## Limitations

Although this dataset addresses a significant gap for Urdu as a low-resource language in OCR research, the following limitations should be noted:

i. The dataset is synthetically generated from digital font renderings. The current font coverage is limited to 201 font styles; while this substantially exceeds existing Urdu character-level benchmarks, the Urdu typography landscape continues to evolve. The proposed structured pipeline supports straightforward dataset expansion as new font styles become available.
ii. The augmentation pipeline simulates a range of real-world printing and scanning conditions; however, the dataset may not fully capture all artifacts present in physically scanned documents such as ink bleed, paper texture, and print registration variations. The dataset can be extended by adding scanned and camera captured character images from real-world scanned documents in future versions to bridge the domain gap between synthetic training data and practical OCR deployment.
iii. The dataset operates at the character level in isolated form, which is suitable for foundational OCR research and benchmarking. Practical Urdu OCR systems typically require recognition at the ligature, word, or line level. Furthermore, Urdu characters in connected text assume positional variants (initial, medial, final forms) and may combine into ligatures not represented in this isolated-character dataset. These contextual shaping complexities, combined with Urdu's bidirectional rendering requirements, present additional challenges for word- and line-level OCR which represents a natural direction for future dataset development.

## Ethics Statement

The authors have read and follow the ethical requirements for publication in Data in Brief and confirming that the current work does not involve human subjects, animal experiments, or any data collected from social media platforms.

All 201 font files used in dataset construction were sourced from publicly available repositories and verified to carry open or freely redistributable licenses compatible with the CC BY 4.0 distribution license applied to this dataset. The font collection spans multiple calligraphic styles including Nastaleeq, Naskh, Kufi, Thuluth, and Ruqaa. Nastaleeq-style fonts constitute the largest proportion of the collection, consistent with its predominance in printed Urdu publications, newspapers, and books. This stylistic distribution reflects real-world Urdu typography and should be considered when applying the dataset to OCR tasks requiring uniform performance across all calligraphic styles.

## Credit Author Statement

**Fauzia Yasir:** Conceptualization, Methodology, Software, Data Curation, Writing – Original Draft, Visualization. **Majida Kazmi:** Conceptualization, Supervision, Validation, Writing – Review & Editing. **Saad Ahmed Qazi:** Project Administration, Visualization, Writing – Review & Editing.

## Data Availability

Mendeley DataFIPU-OCR-CHAR: Font-Invariant Printed Urdu Character Dataset (Original data).

## References

[1] What are the top 200 most spoken languages? Ethnologue. [Online].https://www.ethnologue.com/insights/ethnologue200/ Accessed: October 6, 2025.

[2] Nasir T., Malik M.K. (2024). Efficient CRNN towards end-to-end low resource Urdu text recognition using depthwise separable convolutions and gated recurrent units. Inf. Process. Manag..

[3] Hussain S., Ali S.S., Akram Q.U.A. (2015). Nastalique segmentation-based approach for Urdu OCR. Int. J. Doc. Anal. Recognit..

[4] Sagheer M.W., He C.L., Nobile N., Suen C.Y. (2009). Image Analysis and Processing – ICIAP 2009, LNCS.

[5] Ahmed S.B., Naz S., Swati S., Razzak M.I. (2019). Handwritten Urdu character recognition using one-dimensional BLSTM classifier. Neural Comput. Appl..

[6] S.B Ahmed, S. Naz, S. Swati, & M.I. Razzak, (2017). Handwritten Urdu character recognition using 1-dimensional BLSTM classifier. arXiv:1705.05455. 10.48550/arXiv.1705.05455.

[7] Nasir T., Malik M.K., Shahzad K. (2021). MMU-OCR-21: towards end-to-end Urdu text recognition using deep learning. IEEE Access.

[8] Rahman A., Ghosh A., Arora C., Fink G.A., Jain R., Kise K., Zanibbi R. (2023). Document Analysis and Recognition - ICDAR 2023. ICDAR 2023. Lecture Notes in Computer Science.

[9] Yasir F., Kazmi M. (2025). Acceleration of Urdu optical character recognition on Zynq UltraScale+ MPSoC using deep convolutional neural network. IEEE Access.

[10] LeCun Y., Bottou L., Bengio Y., Haffner P. (1998). Gradient-based learning applied to document recognition. Proc. IEEE.

[11] G. Cohen, S. Afshar, J. Tapson, & A. van Schaik, (2017). EMNIST: An extension of MNIST to handwritten letters. arXiv:1702.05373. 10.48550/arXiv.1702.05373.

[12] The Qt Company (2026). PySide6: qt for Python (Version 6.9.1) [Computer software]. https://doc.qt.io/qtforpython-6/.

[13] Clark J.A., Contributors (2026). Pillow (Version 11.2.1) [Computer software]. Zenodo.

